# Beyond Hydronephrosis: Perirenal Fluid on Screening Ultrasound in Patients With Renal Colic at a Basic Emergency Service

**DOI:** 10.7759/cureus.105166

**Published:** 2026-03-13

**Authors:** Sergio Miravent, Narciso B Molina, Juan Ruiz, Teresa Silva, Carmen J Bermejo

**Affiliations:** 1 Department of Basic Emergency Service, Vila Real de Santo António Health Center, Algarve Local Health Unit, Vila Real de Santo António, PRT; 2 Department of Medical Imaging and Radiotherapy, Higher School of Health Sciences, University of Algarve, Faro, PRT; 3 Department of Critical Care, Algarve University Hospital Centre, Algarve Local Health Unit, Faro, PRT

**Keywords:** hydronephrosis, perinephric fluid, renal colic, triage, ultrasonography, ureterolithiasis

## Abstract

Screening ultrasound has been progressively integrated into the evaluation of renal colic in emergency settings, enabling rapid identification of indirect signs of urinary obstruction, such as hydronephrosis, and supporting clinical triage when laboratory testing is limited. This article reports a case of recurrent severe renal colic refractory to analgesia, in which screening ultrasound demonstrated moderate unilateral hydronephrosis associated with perinephric/perirenal fluid. We discuss the clinical significance of this combined finding in light of the available evidence, acknowledging that the literature remains inconclusive: some studies link perinephric fluid to more severe presentations, whereas others do not show a consistent impact on hard outcomes such as hospitalization, emergency department length of stay, or the need for urologic intervention. In a Basic Emergency Service (BES) setting without immediate access to detailed renal function analysis, the coexistence of moderate hydronephrosis and perinephric fluid, particularly in the context of persistent pain, should be interpreted as a contextual marker of potentially clinically significant obstruction and/or urinary extravasation, warranting a lower threshold for referral for complementary evaluation and specialist decision-making. Finally, we propose a BES-specific decision-support table integrating key clinical, limited laboratory, and ultrasound variables to guide management pathways for renal colic in our local setting.

## Introduction

USG is widely recommended for evaluating suspected renal colic and is frequently used as a first-line screening tool during the initial assessment. It helps identify clinically relevant urinary tract pathology and rapidly answer a practical question: are the findings normal, abnormal, or inconclusive? [1,2] This is particularly relevant in small peripheral emergency settings, where laboratory and imaging resources are limited and specialist support is usually centralized, often requiring transfer. In such contexts, screening ultrasound performed by a trained sonographer can support early decision-making and appropriate referral [3,4].

In our Basic Emergency Service (BES), recurrent presentations with renal colic are common. Previously published data from a small Portuguese BES cohort (n=31) showed that 80% of patients had at least one prior emergency attendance for a similar episode of renal colic. Short-term revisits for renal colic have also been reported in larger studies. In a large all-payer cohort of patients discharged from emergency departments with kidney stones in California, 11% returned within 30 days. These data further support the need for consistent risk stratification and clear referral pathways in the management of renal colic [5,6].

Screening renal and urinary tract ultrasound should include both kidneys and the urinary bladder using standard views in two planes, with a pragmatic focus on hydronephrosis and bladder distension or retention [7]. When stone disease is suspected, an echogenic focus with shadowing should be sought. Color Doppler and the twinkling artifact may support stone detection when direct visualization is limited, and ureteral jets at the ureterovesical junction can provide supportive evidence of ureteral patency [8,9,10]. In the same resource-limited setting, ultrasound may also assist in bladder-related decision-making, including confirming intravesical catheter and balloon position in difficult catheterization [11,12].

Perirenal (perinephric) fluid is another finding that may accompany suspected ureteral obstruction on screening ultrasound. In this setting, the most rupture-prone portion of the collecting system is the renal fornix at the tip of the minor calyces [13]. Although perirenal fluid may be relatively common in renal colic and urolithiasis, its clinical relevance, particularly its implications for decision-making and referral in peripheral emergency settings, remains uncertain and is addressed in the Discussion section. In practice, interpretation of this finding is often not straightforward for non-specialist clinicians and may generate uncertainty regarding its meaning and urgency during the initial assessment.

## Case presentation

A 56-year-old woman, with no known drug allergies and a past medical history of dyslipidaemia treated with a statin, presented to the ED with acute abdominal pain radiating to the right lumbar region, accompanied by vomiting since 3:00 a.m. on the day of admission. She reported a similar episode approximately one month earlier, when she was evaluated in the BES for low back pain in the context of a UTI; symptoms improved, and no further diagnostic work-up was performed.

At triage, the chief complaint was documented as “abdominal pain radiating to the lumbar region associated with vomiting.” The Manchester Triage System “Abdominal Pain” flowchart was applied, and she was assigned an orange priority level (very urgent). Subsequently, on medical evaluation, a urine dipstick test (Combur test) demonstrated significant haematuria (+++). Physical examination revealed marked right costovertebral angle tenderness (positive right renal Murphy sign), with no tenderness on the left side; the remainder of the physical examination was unremarkable. Symptomatic management was initiated with tramadol, metoclopramide, and paracetamol.

**Figure 1:**
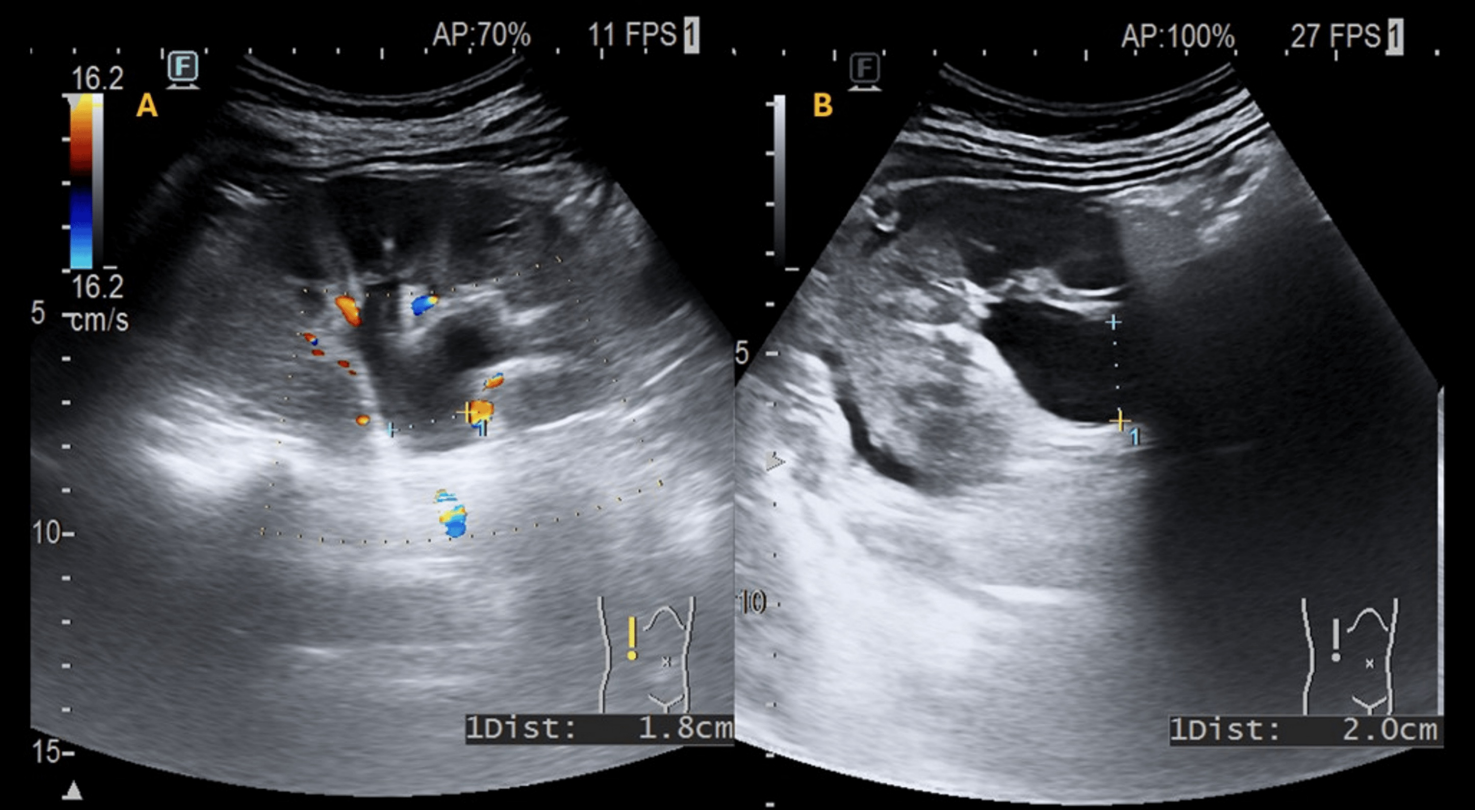
Right renal pelvis measured in two orthogonal planes. Panel A shows a longitudinal renal ultrasound view of the right kidney with pelvicalyceal dilatation; color Doppler is used to confirm mild-to-moderate hydronephrosis. The longitudinal (long-axis) renal pelvis diameter measures 1.8 cm, and in the subsequent Panel B axial view, it measures 2.0 cm, consistent with at least mild-to-moderate hydronephrosis, without sonographic evidence of cortical thinning. Panel B also demonstrates a thin, elongated, crescentic anechoic perirenal fluid collection, seen as a black laminar image along the posterolateral renal margin, interposed between the outer renal cortical contour and the adjacent perirenal interface tissues.

**Figure 2:**
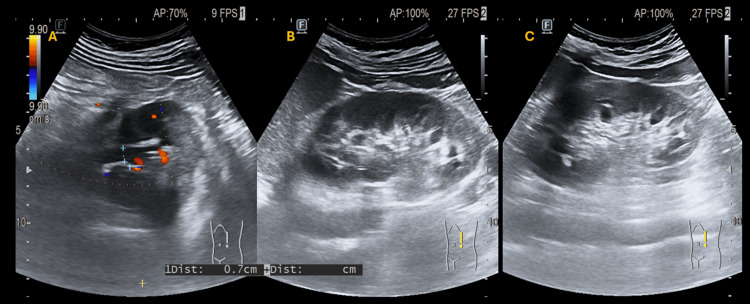
Left kidney seen in one axial image and two longitudinal images. Panel A shows the left renal pelvis on axial view, measuring approximately 7 mm, without calyceal dilatation and with no sonographic evidence of hydronephrosis. Panels B and C show longitudinal ultrasound views of the left kidney, confirming the absence of sonographic hydronephrosis.

Despite completion of the initial therapeutic measures, the patient continued to report persistent pain. At that time, the attending physician requested screening renal ultrasound, depicted in Videos 1-3 and Figures 1-2.

**Video 1:** Biplanar ultrasound views of the right kidney and renal pelvis. Panel A shows a long-axis (lateromedial sweep) view of the right kidney demonstrating pelvicalyceal dilatation consistent with mild-to-moderate hydronephrosis; color Doppler shows no flow within the most anechoic region, confirming fluid within the dilated collecting system. Panel B shows an axial sweep through the renal hilum, depicting the dilated renal pelvis and a peripheral anechoic layer surrounding the kidney and adjacent to the posterolateral renal cortex, consistent with perirenal (perinephric) fluid.

**Video 2:** Two longitudinal ultrasound scans of the right kidney showing the extent of the perinephric fluid collection. Panels A and B show longitudinal ultrasound views of the right kidney demonstrating a thin, elongated, crescentic anechoic fluid collection, appearing as a black laminar image on ultrasound, along the posteroinferior renal margin. The collection is interposed between the outer renal cortical contour and the adjacent perirenal interface tissues, extending from approximately the interpolar region toward the lower pole. Its localized distribution and close apposition to the renal surface favour perirenal/perinephric fluid rather than nonspecific intraperitoneal free fluid. In Panel B, central pelvicalyceal dilatation consistent with mild-to-moderate hydronephrosis is also visible.

**Video 3:** Evaluation of the bladder and right-sided urinary stream patency. Panel A shows the bladder trigone demonstrating focal thickening/bulging at the right ureteral orifice, compatible with reactive oedema in the setting of a possible ureteral calculus impacted at the distal ureterovesical junction (UVJ). An adjacent echogenic focus is present. Concurrently, no right-sided ureteral jet is detected on color Doppler during the urinary stream patency test. Panel B shows color Doppler at the same site demonstrating a twinkling artifact over the echogenic focus, supporting possible distal ureterolithiasis at the right UVJ.

Despite analgesic therapy, the patient’s pain remained severe and refractory, and given the ultrasound findings, which were highly suggestive of right-sided unilateral obstruction, together with the additional concern of a consistent perirenal fluid collection, the patient was referred to the central hospital. In this context, clinicians often question the clinical significance of perirenal fluid. When perirenal fluid is seen together with hydronephrosis, uncertainty may arise regarding the most appropriate next steps. This is understandable, as a clear, standardized management pathway based on combined clinical and ultrasound findings is not available in peripheral emergency settings.

For this reason, we developed and suggest a simplified, optional reference table (Table 1) for clinical consultation. It integrates key clinical and point-of-care data, namely the presence and degree of hydronephrosis, perirenal fluid (absent or present), urinalysis findings, and basic laboratory markers such as the complete blood count. By combining the most common permutations of these variables, the table summarizes what is most likely occurring in each scenario and suggests the most appropriate and probable disposition, such as safe discharge with follow-up, short-interval reassessment, or referral to the central hospital.

**Table 1:** Renal screening ultrasound in suspected renal colic. Locally developed educational table intended to support structured assessment and referral-oriented reasoning in the Basic Emergency Service (BES); it is not a validated standalone clinical decision tool. *Acute flank/lower back pain ± groin radiation ± nausea/vomiting ± haematuria; persistent symptoms despite analgesia or repeated BES visits. This decision-support table is an educational synthesis created by the authors for use in a peripheral emergency setting. Conceptually derived from recommendations of the European Association of Urology and the American College of Radiology, and supplemented by peer-reviewed literature on Doppler ureteral jet evaluation and urinary extravasation, it does not represent an official guideline or consensus statement. Prospective validation is needed before broader recommendation or implementation. Leu: Leukocytes; Nit: Nitrites; CBC: Complete blood count.

Hydronephrosis	Perirenal fluid	Ureteral jets (bladder Doppler)	Urinalysis (Leu/Nit)	CBC (leukocyte count)	Likely interpretation	Suggested management
Absent/mild	Absent	Present and symmetric	Normal	Normal	Uncomplicated renal colic / no significant obstruction	Conservative management (analgesia, discharge instructions, follow-up)
Moderate/severe	Absent	Asymmetric or absent	Normal	Normal	Obstruction likely, even without perirenal fluid	Close observation and consider additional imaging/referral according to the clinical context
Mild/moderate	Present	Present and symmetric	Normal	Normal	Increased intrapelvic pressure / possible contained forniceal rupture with preserved drainage	Conservative management with scheduled clinical reassessment (with awareness of possible complications)
Mild/moderate	Present	Present but asymmetric	Normal	Normal	Partial obstruction	Observation with clinical reassessment; repeat ultrasound and/or laboratory tests if clinically indicated
Moderate/severe	Present	Absent on the symptomatic side	Normal	Normal	Significant obstruction with urinary extravasation	Refer to Urology (CT according to local protocol)
With or without hydronephrosis (often moderate/severe)	Present	Absent	May be normal or abnormal	May be normal or abnormal	Probable urinary tract infection; if obstruction or pressure markers are present, there is a risk of infected obstruction	Urgent referral (high priority; consider CT)
With or without hydronephrosis	Regardless of perirenal fluid status	Regardless of ureteral jet pattern	Leu+ and/or Nit+	± leukocytosis	Infection is likely. In the appropriate clinical context, treat as possible infected obstruction even if ultrasound findings appear reassuring	Urgent escalation of care: prompt Urology involvement, consider urgent CT per protocol, and assess for sepsis/initiate management per local pathway

On arrival at the central hospital, the patient reported right-sided flank pain radiating to the pelvic region, associated with urinary symptoms including dysuria, pollakiuria, and vesical tenesmus, without fever. Initial evaluation documented leukocytosis of approximately 13,000 cells per µL, negative CRP, preserved renal function, and urinalysis positive for leukocytes and blood.

A non-contrast CT scan of the abdomen and pelvis was requested to further characterize the obstruction and assess for complications. The radiologist’s report stated: “… The liver, gallbladder, pancreas, spleen, left kidney, and adrenal glands show no significant abnormalities. A pericentrimetric calculus is identified in the right ureteral meatus, causing ureterohydronephrosis (renal pelvis measuring 2.5 cm in anteroposterior diameter). There is increased attenuation of the perirenal fat, consistent with associated inflammatory changes. No dilated bowel loops are observed. No peritoneal fluid is detected …”

We cannot confirm with 100% certainty that both modalities depict the exact same calculus; however, concordant laterality and the findings on the CT scan strongly corroborate the same location of obstruction. Importantly, as shown in the above-mentioned CT report, there is no mention of additional calculi or of an alternative, more precise stone location beyond what is described.

CT perinephric fat stranding and sonographic perirenal/perinephric fluid should not be considered strictly synonymous findings. Perinephric fat stranding on CT reflects fluid/oedema within the perinephric fat and bridging septa as a secondary sign of obstruction, whereas sonographic perinephric fluid represents a more overt fluid collection around the kidney; nevertheless, both may belong to the same obstructive pathophysiologic spectrum.

Following initial evaluation by the Urology team, medical expulsive therapy was initiated. The patient remained hospitalised for 24 hours for observation, with complete symptom resolution, and was discharged with outpatient follow-up recommended as a precaution.

## Discussion

Perinephric fluid on screening renal ultrasound has been proposed as an indirect sign of increased collecting-system pressure, potentially reflecting a higher degree of obstruction. In support of its clinical relevance, Nadav G et al. found that sonographic perinephric fluid was significantly associated with more severe pain, reflected by higher analgesic requirements and increased morphine use in the ED [14]. Similarly, Cannata D et al. [15] reported that, in ED patients with renal colic and ureterolithiasis, perinephric fluid on renal point-of-care ultrasound was associated with larger stone size (≥5 mm) and a higher likelihood of urologic intervention. These associations suggest that perinephric fluid may co-occur with a cluster of features consistent with more significant obstruction, supporting its potential role as a marker of severity rather than a purely incidental finding.

However, the prognostic value of perinephric fluid remains inconsistent across cohorts. In an ED population, Moretto S et al. [16] found that perinephric fluid was relatively common but was not independently associated with higher rates of urologic intervention, length of stay, or worse clinical outcomes when considered alongside other clinical and imaging variables.

Overall, the current literature supports interpreting perinephric fluid as a potential marker of obstruction severity, but not as a stand-alone predictor of intervention or hospitalization. Accordingly, we suggest a pragmatic interpretation framework, summarized in Table 1, in which perinephric fluid increases vigilance primarily when it coexists with hydronephrosis and concerning clinical or laboratory features. When pain persists, the clinical trajectory remains uncertain, or local diagnostic and monitoring resources are limited, a cautious approach, including referral to a central hospital for further evaluation, may be appropriate.

In our BES, screening renal ultrasound is carried out by radiographers/sonographers who, beyond structured theoretical instruction and supervised hands-on emergency ultrasound training, are committed to maintaining this skill as part of a patient-centred approach to acute care. In practice, clinicians and sonographers involved in emergency ultrasound readily appreciate the qualitative difference between admissions with and without on-shift ultrasound coverage, particularly in terms of earlier clinical clarification that supports patient management and safety. Nevertheless, renal ultrasound remains an inherently operator-dependent examination, and diagnostic performance for hydronephrosis varies with training and experience [17]. In addition, renal ultrasound has important pitfalls that may lead to false-positive interpretation of obstruction. For example, an extrarenal pelvis can mimic hydronephrosis on point-of-care ultrasound; differentiation requires careful assessment in two orthogonal planes, attention to the presence or absence of calyceal dilatation and cortical thinning, and the use of adjunct techniques such as color Doppler when appropriate [18].

## Conclusions

In this case, screening renal ultrasound performed in a peripheral emergency setting by a trained radiographer-sonographer aided the recognition of hydronephrosis and associated perirenal fluid, contributing to clinical assessment and referral. This observation suggests that focused screening ultrasound may have a useful complementary role in selected patients with suspected urinary obstruction, particularly for early diagnostic clarification in settings where immediate imaging options are limited and initial decision-making may otherwise rely mainly on clinical and laboratory assessment. Prospective studies are needed to further evaluate its reproducibility and clinical impact.
